# Cost of London and Glasgow Hospitals Compared

**Published:** 1916-01-01

**Authors:** 


### The Editor's Letter-Box.

### Cost of London and Glasgow Hospitals Compared.

*To the Editor of* THE HOSPITAL.

SIR,—With reference to the letter of Lord Knutsford which you published in your issue of December 25, 1915, it may interest your readers to be reminded that the question of the relative cost of London and Scotch hospitals was fully dealt with in Dr. Mackintosh's paper on the "Control of Hospital Expenditure with Efficiency" and the discussion thereon (see THE HOSPITAL, March 11, 1905, pp. 430 to 435 inclusive). On page 420 of the same number there was an interesting quotation from the *Times*, and certain figures relating to this question are put to the test in THE HOSPITAL of March 18, 1905, pp. 451 and 452. —I am, Sir, yours faithfully, GLASGOW.

